# A Novel Homozygous Frameshift Mutation in *CCN6* Causing Progressive Pseudorheumatoid Dysplasia (PPRD) in a Consanguineous Yemeni Family

**DOI:** 10.3389/fped.2019.00245

**Published:** 2019-06-25

**Authors:** Nagwa E. A. Gaboon, Asia Parveen, Ahmed El Beheiry, Jumana Y. Al-Aama, Mosab S. Alsaedi, Naveed Wasif

**Affiliations:** ^1^Medical Genetics Center, Faculty of Medicine, AinShams University, Cairo, Egypt; ^2^Institute of Molecular Biology and Biotechnology, Center for Research in Molecular Medicine, The University of Lahore, Lahore, Pakistan; ^3^Faculty of Life Sciences, University of Central Punjab (UCP), Lahore, Pakistan; ^4^Department of Radiodiagnosis and Interventional Radiology, Faculty of Medicine, Alexandria University, Alexandria, Egypt; ^5^Department of Genetic Medicine, Faculty of Medicine, King Abdulaziz University, Jeddah, Saudi Arabia; ^6^Princess Al-Jawhara Albrahim Center of Excellence in Research of Hereditary Disorders, King Abdulaziz University, Jeddah, Saudi Arabia; ^7^Institute of Human Genetics, University of Ulm, Ulm, Germany; ^8^Institute of Human Genetics, University Hospital Schleswig-Holstein, Kiel, Germany

**Keywords:** progressive pseudorheumatoid dysplasia (PPRD), consanguinity, novel frameshift mutation, *CCN6*, Nonsense Mediated Decay

## Abstract

**Background:** Progressive pseudorheumatoid dysplasia (PPRD) inherited in an autosomal recessive fashion, is a disabling disease, characterized by platyspondyly, irregularities of the vertebral bodies, narrowing of the intervertebral discs and intraarticular spaces, widening of the epiphysis-metaphysis, polyarthralgia, multiple joint contractures, and disproportionate short stature. A number of studies have been performed on this deformity in various populations around the globe, including the Arab population. Mutations in *CCN6*, located on 6q22, are reported to cause this anomaly.

**Case Presentation:** The present study describes the investigation of a consanguineous family of Yemeni origin. Clinical examination of the patient revealed short stature with progressive skeletal abnormalities, stiffness and enlargement of small joints of the hands along with restriction of movements of proximal interphalangeal (PIP) and distal interphalangeal (DIP) joints with weakness and gait disturbance. Sanger sequencing revealed a novel homozygous frameshift deletion mutation (c.746delT; p.Val249Glyfs^*^10) in *CCN6* which may lead to NMD (Nonsense mediated decay). This mutation expands the spectrum of pathogenic variants in *CCN6* causing PPRD.

## Introduction

Spondylo-epi-metaphyseal dysplasias (SEMDs) are a heterogeneous group of disorders carrying different inheritance patterns (1). SEMDs are described as a set of epiphyseal, metaphyseal, and vertebral abnormalities. SEMDs are diagnosed based on typical skeletal presentations and/or associated extra clinical features of skeletal defects (2, 3). PPRD (OMIM 208230); Spondyloepiphyseal dysplasia tarda with progressive arthropathy (SEDT-PA), is described as a progressive stiffness of joints, remarkably reduced mobility of the cervical spine, and swollen fingers (4). Early development among patients with PPRD is usually normal but during the initial years of childhood the formation of the joint contractures occurs in the hands which spreads to the knees, hips, and the spine (5).

As PPRD is a progressive disorder, all the joints (small and large, including spine) will have progressively limited movement. Stature is usually normal at the onset of the disease but have been reported below percentile 3 at the time of diagnosis in most of the cases (6). Garcia Segarra et al. (6) have also reported that there are variations in early symptoms of the patients but about 50% of the patients had gait anomalies and fatigability. In 30% of the cases the first sign was IPJ (interphalangeal joints) swelling and some degree of knee deformity in about 20% of the cases while pain was reported as the initial symptom in 15% of the patients (6). Radiological surveys indicate compressed vertebral bodies, defects in ossifications, and malformed acetabular portion of pelvis (7). PPRD has an autosomal recessive mode of inheritance which seems to be more frequent in the Middle East and Gulf states because of the high consanguinity rate and large family size, with an incidence of ~1:1,000,000 in Arab countries, (7, 8), however a few cases from non-Mediterranean origin (Germany, United Kingdom, Belgium, Italy, Poland, France UK, USA, Morocco, Ecuador, Japan, India, Pakistan, and China) have been reported as well (5, 6, 9).

PPRD phenotype is often confused with skeletal deformities of ankylosing spondylitis, rheumatoid arthritis (RA), and mucopolysaccharidosis (1, 6) but the characteristic features of RA, such as the destruction of bones, is missing in PPRD (7). Though PPRD is clinically indistinguishable from juvenile idiopathic arthritis (JIA) (7), the results regarding RA factor and inflammation during diagnostic testing are always negative and PPRD patients also show a reduced response to antirheumatic drugs (5, 7).

PPRD is caused by mutations in cellular communication network factor 6 (CNN6), previously known as Wnt1 inducible signaling pathway protein 3 (WISP3), which maps to chromosome 6q22 (10), and encodes a connective tissue growth factor involved in cell growth and differentiation (11). The human cartilage homeostasis and bone growth are highly dependent on the expression of CCN6 (12, 13).

Two homologs CCN4 and CCN5 (also known as WISP1, WISP2) of CCN6 have been described in the literature, which are expressed in WNT1 transformed cell (14, 15). The role of WNT-signaling proteins in cell fate determination and the regulation of cell morphology and proliferation is extraordinary (15–18). CCN6, along with its orthologs (CCN4, CCN5), shows a differential expression in primary human colon cancer (14, 15). Hurvitz et al. pointed out that CNN6 might have a consistent role in human skeletal homeostasis and the regulation of skeletal development in adults (15).

In the present study, we clinically diagnosed PPRD in a Yemeni patient, born from a consanguineous marriage. Sanger sequencing of coding regions of *CCN6* has revealed a novel deletion mutation (c.746delT; p.Val249Glyfs^*^10) in this patient.

## Case Presentation

### Methods

**Figure 1:**
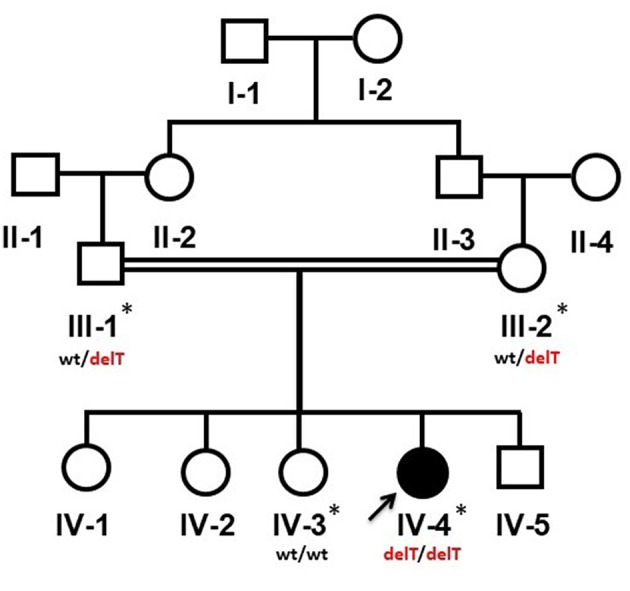
Pedigree of a Yemeni family segregating an autosomal recessive PPRD. Arrow indicates the index patient (IV-4). The samples which were available for the genetic analysis are marked with asterisks (*). wt/delT represents the heterozygous genotype of the carriers, wt/wt represents the wild type genotype of an unaffected member while delT/delT represents the homozygous deletion in the affected member of the family.

The study was conceived according to the principals of the Declaration of Helsinki. Both adult and minor participants were completely informed about the clinical procedures and molecular diagnosis. Informed written consents of parents were obtained on the institutional patient recruitment and consent proformas and research protocols were approved under the reference number 24/14 by the medical ethics and research committee of King Abdulaziz University, Jeddah, Saudi Arabia. Affected member (IV-4) underwent thorough clinical, radiological and other relevant laboratory investigations. Venous blood samples were collected from affected (IV-4) and available unaffected individuals (III-1, III-2, IV-3) of the family (Figure 1). Genomic DNA was extracted following a standard protocol.

### Sanger Sequencing

Chain termination DNA sequencing technique was used for the identification of pathogenic variant in *CCN6* and co-segregation analysis in the family. The University of California Santa Cruz (UCSC) genome database browser (http://genome.ucsc.edu/cgi-bin/hgGateway) was used to extract the *CCN6* genomic transcript (NM_198239.1) and the mutation nomenclature of *CCN6* is also based on this transcript. Primers (Table 1) for the coding regions of *CCN6* were designed using AmplifX v1.5.4 software (http://crn2m.univ-mrs.fr/pub/amplifx) and PCR amplification was carried out to amplify the sequences of interest. Exo-Sap protocol (https://www.thermofisher.com) was used for the cleanup of PCR products. Sanger sequencing was performed on an ABI 3730 genetic analyzer with BigDye chemistry v3.1. SeqMan Pro (DNASTAR, Inc., Madison, WI, UK) was used to align the chromatograms with the reference sequence. Genome aggregation database (gnomAD, http://gnomad-old.broadinstitute.org/) was consulted to verify the allele frequency of the pathogenic variant.

**Table 1:** Primer sequences of all exons of *CCN6* used for the PCR amplification.

**Nr**.	**Gene, Exon**	**Tm**	**Sequences**
1	*CCN6*_Exon 1-F	62.8°C	ctcactgcgaaggcaggttatt
2	*CCN6*_Exon 1-R	64.7°C	ctgtgccaggctgtgcctta
3	*CCN6*_Exon 2-F	61.4°C	aacagtcttggaggctgagtga
4	*CCN6*_Exon 2-R	60.3°C	ggcttctgaatgtgtgtaagca
5	*CCN6*_Exon 3-F	57.9°C	acctttctacagatgtcctgtga
6	*CCN6*_Exon 3-R	55.7°C	tagtagagcttcctgacctagtagat
7	*CCN6*_Exon 4-F	63.0°C	attcagaggacaggcaaagcag
8	*CCN6*_Exon 4-R	64.1°C	tccccacaacatgcagttaatacc
9	*CCN6*_Exon 5-F	58.7°C	cttaagggtaaagagagtgctgg
10	*CCN6*_Exon 5-R	58.4°C	tgcttagatggtaacaaatgtctg

*Tm, melting temperature; ^0^C, centigrade; F, Forward; R, Reverse*.

## Results

A 13-year-old patient (IV-4) was referred to the genetic clinic, King Abdulaziz University Hospital, Jeddah, for the genetic consultation. After review of the family history, clinical symptoms, and laboratory investigations of the patient, there were several clues which guided us to the diagnosis of PPRD in this patient rather than JIA. This included the consanguinity of the parents, the absence of systemic involvement and poor response to antirheumatic drugs. The radiological findings, especially the absence of erosion, were the key indicators. The family was subjected to DNA sequencing of *CCN6* to know the genetic cause of this anomaly and for appropriate genetic counseling.

### Clinical Findings

The 13-year-old female (IV-4) is the 5th offspring of a consanguineous Yemeni couple (III-1, III-2) (Figure 1). She was born to a healthy 33-year-old mother (III-2) and a 37-year-old father (III-1) as a result of an uncomplicated pregnancy a following normal vaginal delivery. Her birth parameters including length, weight and skull circumference were within normal range in reference to her gestational age in weeks. The family history was unremarkable, and other siblings were healthy.

**Figure 2:**
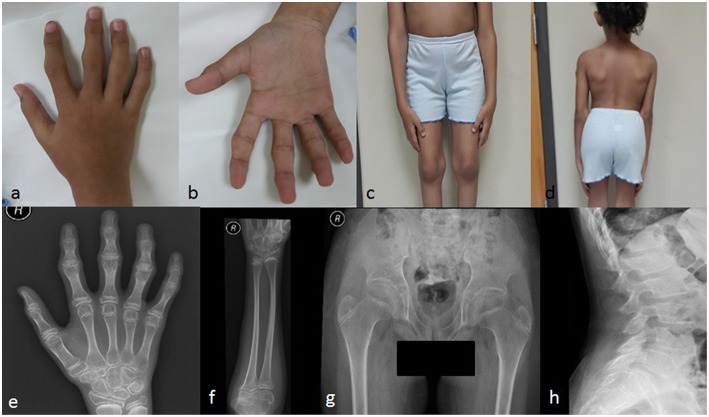
Clinical pictures of the patient IV-4 **(a)** showing camptodactyly of all fingers, with prominent PIP and DIP joints **(b)** represents the inability of the patient to make a fist **(c)** shows the prominent knee joints **(d)** shows short trunk. Radiological findings of the patient IV-4. **(e)** an X-rays of the right hand shows the irregular articular surface of the PIP joints with relative decrease in joint space and enlargement of the epi-metaphysis of the interphalangeal joints **(f)** shows elbow joints with prominent capitulum and trochlea of the distal humerus and irregularity of the articular surface **(g)** shows hips with bilateral fine irregularities of articular surfaces **(h)** shows the anterior beaking of the vertebral bodies with mild form of platyspondyly.

The patient displayed normal development until the age of four years. In her fifth year, she started complaining of progressive stiffness, swelling and restriction of movements of proximal interphalangeal (PIP), distal interphalangeal (DIP), and metacarpophalangeal (MCP) joints with an inability to make a fist (Figures 2a,b). The progressive restriction of movements of the elbow, knee, hip, ankle, shoulder, and wrist, associated with progressive difficulty in walking and abnormal gait was observed. She exhibited no inflammatory signs in her joints.

She had a disproportionate short trunk and short stature; on assessment at the age of thirteen, her height was = −3.7SD centile (130 cm); skull circumference was at 25th percentile (52 cm,) and weight was in 5th percentile (27 kg). On physical examination, she had prominent knobby appearing interphalangeal joints (PIP and DIP), fixed flexion deformity of the PIP, and MCP joints of the hands with an inability to make a fist (Figure 2b), along with a limited range of active and passive movements mainly at intraphalangeal, elbow, and knee joints (Figure 2c). Beaking of the vertebrae was another evident feature (Figure 2d). Abduction of the hips was slightly restricted. There was restricted flexion and extension of the neck. No tenderness was experienced in the joints during examination. There was no facial dysmorphism, teeth abnormality or corneal cloudiness. The patient was cooperative with normal mental health.

### Investigations

Cytogenetic analysis of the patient (IV-4) revealed a 46, XX karyotype. Rheumatoid factor, antinuclear antibody, and anti-dsDNA tests were negative. Erythrocyte sedimentation rate, C-reactive protein, complete blood count, coagulation profile, urea and electrolytes, thyroid function tests, parathyroid hormone, and liver enzymes were all within normal range. Bone Marrow Density was −2SD for her age. N-acetylgalactoseamine-6-sulfate sulfatase and arylsulfatase-B were within normal range. The echocardiogram and abdominal ultrasound were unremarkable.

### Radiological Examination

Radiological examination revealed a diffuse decreased density of the visualized bones, irregular articular surface of the PIP joints of hands and feet with relative decrease in joint space and enlargement of the epi-metaphysis of the interphalangeal joints (Figure 2e). This was associated with prominent distal epiphysis of the radius and ulna (Figures 2e,f). Regarding elbow joints, there were prominent capitulum and trochlea of the distal humerus and irregularity of the articular surface (Figure 2f). Hips showed bilateral fine irregularities of articular surfaces (Figure 2g). An X-ray of the spine showed anterior beaking of the lumbar vertebral bodies, mild irregularities of the anterior aspect of the superior end plates along with a mild form of platyspondyly involving most of the examined vertebrae (Figure 2h).

### Mutation Identification

**Figure 3:**
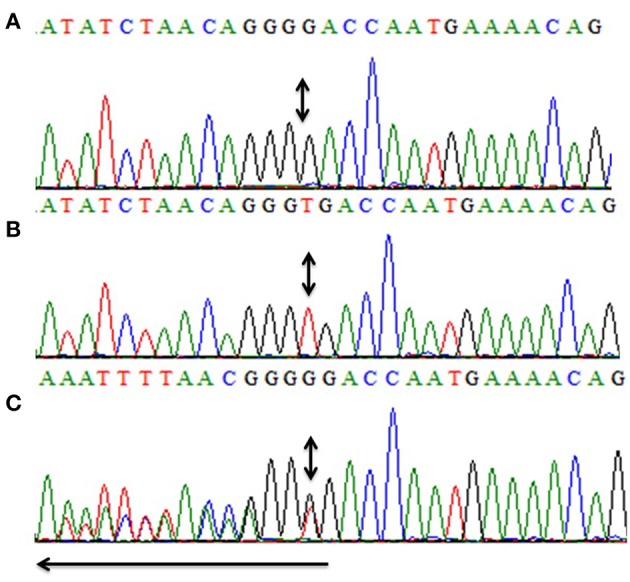
Partial sequence of exon-4 of *CCN6* for the presentation of the novel homozygous frameshift variant (c.746delT, p.V249Gfs*10). Panel **(A)** shows the nucleotide sequence of the affected individual (IV-4) with the homozygous mutation, **(B)** represents the nucleotide sequence of an unaffected individual (IV-3), and **(C)** represents the nucleotide sequence of a heterozygous carrier (III-1). Arrow represents the direction of the sequence while the double-headed arrows represent the position of the homozygous change in all three panels **(A–C)**.

All five exons of *CCN6* were PCR amplified in the affected individual (IV-4) DNA. Sanger sequencing analysis revealed a novel homozygous deletion mutation (c.746delT; p.Val249Glyfs^*^10) in exon-4 of *CCN6* (Figure 3). Considering that the identified variant is pathogenic, Sanger sequencing of both parents (III-1, III-2) and an unaffected sibling (IV-3) was carried out using the same set of primers for exon-4 (Table 1), which confirmed the co-segregation of this variant within the family. The allele frequency of this variant was not found in the Genome Aggregation Database (gnomAD) (http://gnomad.broadinstitute.org/gene/ENSG00000112761). An online prediction algorithm, MutationTaster (http://www.mutationtaster.org) has predicted this variant as a frameshift mutation likely to suffer Nonsense Mediated Decay (NMD). This variant has been submitted to ClinVar database (https://submit.ncbi.nlm.nih.gov/clinvar/) under an accession ID SCV000902272.

## Discussion

Here, we report a consanguineous Yemeni family with one affected member (IV-4) showing typical features of PPRD. The clinical laboratory findings coincided with the apparent clinical phenotypes. The thorough examination of parents (III-1, III-2) and an unaffected brother (IV-3) revealed the absence of PPRD symptoms. Sanger sequencing revealed a homozygous deletion mutation (c.746delT; p.Val249Glyfs^*^10) in *CCN6* which co-segregates within the family. Previously, *CCN6* gene mutations have been reported in PPRD patients from various geographical regions of the world.

*CCN6* encodes a protein of 372 amino acids which is composed of a signal peptide domain encoded by exon-1 and four conserved cysteine-rich domains; IGFBP N-terminal (Insulin-like growth factor binding protein N-terminal) domain, vWFC (von Willebrand factor type-C module) domain, TSP type-1 (thrombospondin) domain, CTCK (C-terminal cystine knot) domain, encoded by exon-2, 3, 4, and 5 of *CCN6*, respectively (15). Missense, nonsense, deletions, insertions and splice site mutations have been reported in this gene causing PPRD phenotype (HGMD, Professional, 2018.3; http://www.hgmd.cf.ac.uk/ac/gene.php?gene=CCN6).

Connective tissue growth factor/cysteine-rich protein 61/nephroblastoma overexpressed (CNN) family member CCN6 is a WNT-signaling pathway protein which plays a vital role in the expression regulation of a transcriptional factor SOX9, collagen II, and aggrecan in chondrocyte cell lines (13, 19). CCN6 plays another role in controlling the activity of superoxide dismutase. The number of reactive oxygen species is increased in the absence of CCN6 expression (6, 20). A study on a single PPRD affected individual conducted by Zhou et al. (21) has provided information about the increased cell proliferation and abnormal matrix metalloproteinase processing in human chondrocytes (21). Certain *in vivo* experiments in zebrafish have established the role of CCN6 in the development of cartilages. It has been proposed that the malfunction of signaling pathways governed by bone morphogenetic protein (BMP) and/or Wnt-signaling protein, in the case of a nonfunctional mutant CCN6, may lead to human cartilage development failure in PPRD patients (22). However, the causative pathomechanism of malformation of cartilage in human PPRD patients is still not well-elaborated (6).

In the present study, we have identified a novel homozygous frameshift variant (c.746delT; p.Val249Glyfs^*^10) in *CCN6* causing PPRD in a Yemeni patient. In a review article, Hentze and Kulozik, have explained that the imperfect messages which are the products of frameshift mutations are eliminated by conserved mammalian surveillance mechanisms like NMD (23). MutationTaster (http://www.mutationtaster.org/) has also predicted that this novel variant may lead to frameshift and eventually to NMD, which is anticipated to cause PPRD in our patient.

## Conclusion

The present work aims to report a clinical study of a Yemeni patient who is a product of a consanguineous union and the identification of pathogenic mutation causing PPRD phenotype. Our data is novel and increases the mutational spectrum of *CCN6* gene.

## Data Availability

No datasets were generated or analyzed for this study.

## Ethics Statement

Informed written consents were obtained from parents about the clinical and molecular diagnosis and for publishing the clinical photographs and mutation data. The study was approved under the reference number 24/14 by the medical ethics and research committee of King Abdulaziz University, Jeddah, Saudi Arabia.

## Author Contributions

NG, MA, and JA-A enrolled the patients, made the clinical diagnosis, and wrote the clinical synopsis. AE did radiological analysis and wrote a report about that. NW and AP designed the study and wrote the initial draft of this manuscript. NW contributed to Sanger sequencing. NW, AP, and NG contributed to critical revisions of the manuscript. All authors have thoroughly reviewed and approved the final draft.

### Conflict of Interest Statement

The authors declare that the research was conducted in the absence of any commercial or financial relationships that could be construed as a potential conflict of interest.

## References

[1] Gun K Uludag M Unalan H Mogulkoc N Battal H Sucuoglu H. A 14-year-old girl with Smith-McCort dysplasia misdiagnosed as seronegative juvenile idiopathic arthritis. Int J Rheum Dis. (2012) 15:e55–7. 10.1111/j.1756-185X.2011.01690.x 22709503

[2] Unger S Superti-Furga A Rimoin DL A Diagnostic Approach to Skeletal Dysplasias. Pediatric bone. Elsevier Science (2003). 10.1016/B978-012286551-0/50018-X

[3] Cormier-Daire V. Spondylo-epi-metaphyseal dysplasia. Best Pract Res Clin Rheumatol. (2008) 22:33–44. 10.1016/j.berh.2007.12.009 18328979

[4] Delague V Chouery E Corbani S Ghanem I Aamar S Fischer J Molecular study of WISP3 in nine families originating from the Middle-East and presenting with progressive pseudorheumatoid dysplasia: identification of two novel mutations, and description of a founder effect. Am J Med Genet Part A. (2005) 138:118–26. 10.1002/ajmg.a.30906 16152649

[5] Yan W Dai J Xu Z Shi D Chen D Xu X. Novel WISP3 mutations causing progressive pseudorheumatoid dysplasia in two Chinese families. Human Genome Variat. (2016) 3:16041. 10.1038/hgv.2016.41 28018607 PMC5143363

[6] Garcia Segarra N Mittaz L Campos-Xavier AB Bartels CF Tuysuz B Alanay Y. The diagnostic challenge of progressive pseudorheumatoid dysplasia (PPRD): a review of clinical features, radiographic features, and WISP3 mutations in 63 affected individuals. Am J Med Genet C Semin Med Genet. (2012) 160C:217–229. 10.1002/ajmg.c.31333 22791401

[7] Spranger J Albert C Schilling F Bartsocas C Opitz JM. Progressive pseudorheumatoid arthropathy of childhood (PPAC): a hereditary disorder simulating juvenile rheumatoid arthritis. Am J Med Genet Part A. (1983) 14:399–401. 10.1002/ajmg.1320140224 6837637

[8] Teebi A Al Awadi S. Spondyloepiphyseal dysplasia tarda with progressive arthropathy: a rare disorder frequently diagnosed among Arabs. J Med Genet. (1986) 23:189. 10.1136/jmg.23.2.189-a 3712405 PMC1049586

[9] Pode-Shakked B Vivante A Barel O Padeh S Marek-Yagel D Veber A. Progressive Pseudorheumatoid Dysplasia resolved by whole exome sequencing: a novel mutation in WISP3 and review of the literature. BMC Med Genet. (2019) 20:53. 10.1186/s12881-019-0787-x 30922245 PMC6439983

[10] Torreggiani S Torcoletti M Campos-Xavier B Baldo F Agostoni C Superti-Furga A. Progressive pseudorheumatoid dysplasia: a rare childhood disease. Rheumatol Int. (2019) 39:441–52. 10.1007/s00296-018-4170-6 30327864

[11] Rachfal AW Brigstock DR. Structural and functional properties of CCN proteins. Vitamins Hormones. (2005) 70:69–103. 10.1016/S0083-6729(05)70003-0 15727802

[12] Davis L Chen Y Sen M. WISP-3 functions as a ligand and promotes superoxide dismutase activity. Biochem Biophys Res Commun. (2006) 342:259–65. 10.1016/j.bbrc.2006.01.132 16480948

[13] Liu L Li N Zhao Z Li W Xia W. Novel WISP3 mutations causing spondyloepiphyseal dysplasia tarda with progressive arthropathy in two unrelated Chinese families. Joint Bone Spine. (2015) 82:125–8. 10.1016/j.jbspin.2014.10.005 25553839

[14] Pennica D Swanson TA Welsh JW Roy MA Lawrence DA Lee J. WISP genes are members of the connective tissue growth factor family that are up-regulated in wnt-1-transformed cells and aberrantly expressed in human colon tumors. Proc Natl Acad Sci USA. (1998) 95:14717–22. 10.1073/pnas.95.25.14717 9843955 PMC24515

[15] Hurvitz JR Suwairi WM Van Hul W El-Shanti H Superti-Furga A Roudier J. Mutations in the CCN gene family member WISP3 cause progressive pseudorheumatoid dysplasia. Nat Genet. (1999) 23:94. 10.1038/12699 10471507

[16] Erlebacher A Filvaroff EH Gitelman SE Derynck R. Toward a molecular understanding of skeletal development. Cell. (1995) 80:371–8. 10.1016/0092-8674(95)90487-5 7859279

[17] Parr BA Avery EJ Cygan JA McMahon AP. The classical mouse mutant postaxial hemimelia results from a mutation in theWnt7a gene. Dev Biol. (1998) 202:228–34. 10.1006/dbio.1998.9007 9769174

[18] Dale CT. Signal transduction by the Wnt family of ligands. Biochem. J. (1998) 329:209–23. 10.1042/bj3290209 9425102 PMC1219034

[19] Sen M Cheng YH Goldring MB Lotz MK Carson DA. WISP3-dependent regulation of type II collagen and aggrecan production in chondrocytes. Arthr Rheum. (2004) 50:488–97. 10.1002/art.20005 14872491

[20] Miller DS Sen M. Potential role of WISP3 (CCN6) in regulating the accumulation of reactive oxygen species. Biochem Biophys Res Commun. (2007) 355:156–61. 10.1016/j.bbrc.2007.01.114 17286957

[21] Zhou H-D Bu Y-H Peng Y-Q Xie H Wang M Yuan L-Q. Cellular and molecular responses in progressive pseudorheumatoid dysplasia articular cartilage associated with compound heterozygous WISP3 gene mutation. J Mol Med. (2007) 85:985–96. 10.1007/s00109-007-0193-2 17483925

[22] Nakamura Y Weidinger G Liang JO Aquilina-Beck A Tamai K Moon RT. The CCN family member Wisp3, mutant in progressive pseudorheumatoid dysplasia, modulates BMP and Wnt signaling. J Clin Invest. (2007) 117:3075–86. 10.1172/JCI32001 17823661 PMC1964511

[23] Hentze MW Kulozik AE. A perfect message: RNA surveillance and nonsense-mediated decay. Cell. (1999) 96:307–10. 10.1016/S0092-8674(00)80542-5 10025395

